# Evaluation of anthracycline-induced subclinical LV dysfunction by using myocardial composite index and two-dimension speckle tracking echocardiography technique

**DOI:** 10.3389/fcvm.2022.936212

**Published:** 2022-08-11

**Authors:** Jiabao Zhu, Shuhui Xie, Hanzhen Ji, Xingxing Gu, Jing Wu

**Affiliations:** ^1^Department of Ultrasound, Nantong Third People’s Hospital, Nantong University, Nantong, China; ^2^Department of Library and Information Science, Nantong Third People’s Hospital, Nantong University, Nantong, China

**Keywords:** speckle tracking technique, myocardial composite index, anthracycline chemotherapy, subclinical LV dysfunction, left ventricular function

## Abstract

**Objective:**

To obtain various myocardial strain parameters by using two-dimension speckle tracking echocardiography (2D-STE) technique, calculate the myocardial composite index (MCI) which combines the global longitudinal strain (GLS) of left ventricle and the left ventricular twist (LVtw), and evaluate their diagnostic efficacies for subclinical left ventricular (LV) dysfunction in patients undergoing anthracycline chemotherapy.

**Methods:**

A total of 35 female breast cancer patients, who underwent postoperative chemotherapy in the Department of Thyroid and Breast Surgery of Nantong Third People’s Hospital from September 2018 to December 2019 and had successful follow-up, were included into the chemotherapy group, and the patients were evaluated respectively at baseline and in early, interim and later chemotherapy stages according to the course of chemotherapy; in addition, 30 healthy women undergoing physical examination during the same period were included into the control group. In different chemotherapy stages, the data such as left ventricular end diastolic diameter (LVEDD), left ventricular end systolic diameter (LVESD), interventricular septal thickness (IVST), left ventricular posterior wall thickness (LVPWT) and left ventricular ejection fraction (LVEF) were collected by using conventional echocardiography, and various myocardial strain parameters such as GLS, global radial strain (GRS), global circumferential strain(GCS) and LVtw were measured using 2D-STE, and then MCI was calculated. Receiver operating characteristic (ROC) curve analysis was performed to evaluate the application values of various parameters in the diagnosis of early cardiotoxicity.

**Results:**

There was a difference in MCI between patients at baseline and in the early chemotherapy stage; there were differences in GLS, LVtw and MCI between patients at baseline and in the interim chemotherapy stage; there were differences in four parameters such as MCI, GLS, LVtw and GCS between patients at baseline and in the later chemotherapy stage; The AUC of MCI was 0.915, when the cutoff value was –210.89 (%×°), the sensitivity and specificity were 84.37% and 90.41%, respectively.

**Conclusion:**

MCI combines the longitudinal and torsional motions of myocardium, and thus has a better diagnostic value for early detection of subclinical LV dysfunction caused by anthracycline chemotherapy drugs compared with strain parameters in a single direction.

## Introduction

The anthracycline chemotherapy drugs are widely used in the treatment of hematological and solid tumors. The anthracycline-based chemotherapy regimen is a classic first-line regimen for the treatment of breast cancers. However, anthracycline drugs have side effects such as bone marrow suppression and cardiotoxicity, and the cardiotoxicity will be gradually aggravated and even irreversible along with the increase of dosage of anthracycline drugs and irreversible at the same time (1). A number of studies have confirmed that the patients may suffer from organic myocardial injury when they firstly use anthracycline drugs (2). Therefore, clinicians should pay great attention to the early detection of cardiotoxicity when using the anthracycline drugs. However, the myocardial injury is often underestimated according to left ventricular ejection fraction (LVEF), and the patients with normal LVEF may also have subclinical LV dysfunction. Therefore, LVEF is not sensitive enough for early detection of early subclinical LV dysfunction (3). The speckle-tracking echocardiography (STE) can be used to quantitatively analyze the movement and changes of myocardium through tracking the movement trajectory of myocardial echo spots on two-dimensional (2D) images, and this is currently a research hotspot in the diagnosis of early cardiac injury. However, two-dimension speckle-tracking echocardiography (2D-STE) technique can only analyze single parameters such as global longitudinal strain (GLS), global circumferential strain (GCS), global radial strain (GRS) and left ventricular twist (LVtw) of the regional myocardium. Myocardial composite index (MCI) can combine left ventricular GLS with LVtw, which can more comprehensively reflect the movement and changes of left ventricular myocardium in the three-dimensional (3D) directions. In this study, the effect of anthracycline drugs on myocardial movement in patients undergoing breast cancer chemotherapy was analyzed by using STE technique, and the application values of various parameters of STE technique in the diagnosis of early cardiotoxicity were evaluated.

## Materials and methods

### Study subjects

Methods: A total 35 female patients, who underwent chemotherapy after breast cancer surgery in the Department of Thyroid and Breast Surgery, Nantong Third People’s Hospital from September 2018 to December 2019 and had successful follow-up, were included into the chemotherapy group, and the patients were evaluated respectively at baseline and in the early (after 2 chemotherapy cycles), interim (after 4 chemotherapy cycles) and later (after 6 chemotherapy cycles) chemotherapy stages according to the course of chemotherapy. In addition, 30 healthy women undergoing physical examination in the hospital during the same period were included into the control group. Inclusion criteria: (1) all patients received anthracycline-based chemotherapy (FAC protocol: fluorouracil 500 mg/m^2^ iv d1, d8; adriamycin 50 mg/m^2^ iv d1; cyclophosphamide 500 mg/m^2^ iv d1; 21 days as a cycle)regimen and completed a total of 6 cycles of chemotherapy; (2) no obvious abnormalities were found in the electrocardiograms of patients before chemotherapy; (3) the patients with basic diseases such as hypertension, diabetes and coronary heart disease were excluded; and (4) the patients had no obvious abnormalities in routine blood biochemical indexes. A total of 50 patients were collected, of whom, 35 were followed up successfully and finally included into the chemotherapy group. The patients aged from 30 to 68 years old, with an average age of (51.66 ± 9.32) years old. The patients at baseline underwent routine echocardiography and 2D-STE at one day before chemotherapy, and the patients in other stages underwent routine echocardiography and 2D-STE at 20 days after chemotherapy. Images were collected and relevant parameters were recorded. All subjects signed an informed consent form, and this study was approved by the ethics committee of our hospital.

### Instruments and methods

**FIGURE 1:**
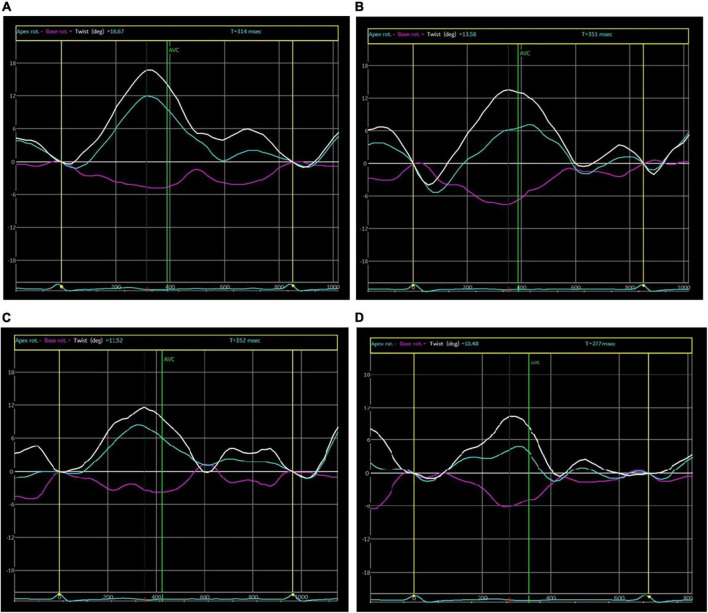
Curve of left ventricular twist. **(A)** at baseline; **(B)** after 2 chemotherapy cycles; **(C)** after 4 chemotherapy cycles; **(D)** after 6 chemotherapy cycles.

VIVID E9 color Doppler ultrasonic instrument of GE Company was adopted, which was equipped with M5S probe with a frequency of 1.5–4.3 MHz and EchoPAC offline workstation. Under the condition of calm breathing, the subjects were placed in a proper left lateral position and connected to an ECG monitor at the same time. The interventricular septum thickness (IVST) at end-diastole, left ventricular end diastolic dimension (LVEDD), left ventricular posterior wall thickness (LVPWT) at end-diastole and left ventricular end systolic diameter (LVESD) were measured with a probe on the long axial section of the left parasternal ventricle. LVEF was detected by using the biplane modified Simpson’s method, the 2D images of three consecutive cardiac cycles were scanned respectively from the short axis views at left ventricular mitral valve annulus, papillary muscle and apical levels, apical four-chamber view, two-chamber view and three-chamber view, which were stored in the DICOM format, then imported into EchoPAC offline workstation and played back frame-by-frame; the myocardial area was manually traced, and the software automatically divided the left ventricular myocardium into 17 segments, and displayed the strain-time curve of each segment and the corresponding bull’s eye diagram. GLS, GCS, GRS and LVtw (Figure 1) were measured. MCI was calculated according to the following formula: MCI = GLS × LVtw (unit:%×°).

### Statistical analysis

SPSS 17.0 software was used for statistical analysis, the measurement data in each group were expressed as mean ± standard deviation and tested for normal distribution and homogeneity of variance. *t*-test or one-way analysis of variance (ANOVA) was used to compare the differences between the groups. A bivariate correlation analysis was performed using Pearson correlation analysis. The receiver operating characteristic (ROC) curves of all global strain parameters were drawn to evaluate their diagnostic values for subclinical LV dysfunction, and the area under the curve (AUC) and the optimal cutoff value were determined.

## Results

### Comparison of various indexes between the control group and the patients at baseline

Table 1 showed that there were no significant differences in various indexes between the control group and the patients at baseline (all *P* > 0.05).

**TABLE 1:** Comparison of various indexes between the control group and the patients at baseline ($\bar{x}$ ± s).

Indexes	Control group	Patients at baseline	*t*	*P*
Age (years)	47.1 ± 9.36	51.66 ± 9.32	–1.942	0.057
LVEDD (mm)	43.55 ± 3.93	43.71 ± 3.44	–0.176	0.861
LVESD (mm)	28.00 ± 2.70	27.83 ± 2.42	0.268	0.790
IVST (mm)	8.13 ± 0.86	8.33 ± 0.81	–0.976	0.333
LVPWT (mm)	8.30 ± 0.70	8.21 ± 0.81	0.447	0.656
LVEF (%)	65.00 ± 3.87	65.83 ± 3.17	–0.942	0.350
GRS (%)	48.19 ± 3.11	47.47 ± 1.96	1.087	0.283
GCS (%)	–18.94 ± 1.77	–19.04 ± 1.7	0.229	0.820
GLS (%)	–19.41 ± 1.68	–18.73 ± 1.41	–1.764	0.083
LVtw (°)	14.58 ± 1.46	14.94 ± 1.05	–1.160	0.250
MCI (%×°)	–271.72 ± 33.51	–280.24 ± 32.62	1.027	0.308

P < 0.05 indicated a statistically significant difference.

### Comparison of various indexes in the chemotherapy group among different chemotherapy stages

Table 2 indicated that the patients in chemotherapy group showed no significant differences in LVEDD, LVESD, IVST, LVPWT, LVEF and GRS among different chemotherapy stages (all *P* > 0.05). The MCI in the early chemotherapy stage was higher than that at baseline, the GLS and MCI in the interim chemotherapy stage were higher than those at baseline, and LVtw in the interim chemotherapy stage was lower than that at baseline. GCS, GLS and MCI in the later chemotherapy stage were higher than those at baseline, and LVtw in the later chemotherapy stage was lower than that at baseline.

**TABLE 2:** Comparison of various indexes in the chemotherapy group among different chemotherapy stages ($\bar{x}$ ± s).

Item	Baseline	Early chemotherapy stage	Interim chemotherapy stage	Later chemotherapy stage	*F*	*P*
LVEDD (mm)	43.71 ± 3.44	43.74 ± 3.22	43.77 ± 3.39	43.49 ± 4.09	0.047	0.986
LVESD (mm)	27.83 ± 2.42	27.71 ± 2.8	28.6 ± 2.69	28.26 ± 3.29	0.730	0.536
IVST (mm)	8.33 ± 0.81	8.37 ± 0.77	8.21 ± 0.83	8.45 ± 0.79	0.529	0.663
LVPWT (mm)	8.21 ± 0.81	8.24 ± 0.78	8.25 ± 0.7	8.44 ± 0.63	0.708	0.549
LVEF (%)	65.83 ± 3.17	65.66 ± 3.11	64.43 ± 3.3	63.77 ± 5.79	2.131	0.099
GRS(%)	47.47 ± 1.96	46.72 ± 1.86	46.35 ± 1.90	45.63 ± 1.77	2.504	0.062
GCS(%)	–19.04 ± 1.70	–17.93 ± 2.90	–17.72 ± 2.93	–16.34 ± 2.57*	6.492	<0.001
GLS(%)	–18.73 ± 1.41	–18.04 ± 1.27	–16.46 ± 1.64*	–15.87 ± 1.48*	24.54	<0.001
LVtw(°)	14.94 ± 1.05	14.42 ± 0.95	13.58 ± 0.78*	12.36 ± 0.76*	49.15	<0.001
MCI(%×°)	–280.24 ± 32.62	–250.41 ± 28.15*	–224.13 ± 24.62*	–196.13 ± 21.41*	62.63	<0.001

*Compared with the patients at baseline, P < 0.05 indicated a statistically significant difference.

### Analysis on the correlations of the cumulative dose of adriamycin with global radial strain, global circumferential strain, global longitudinal strain, left ventricular twist and myocardial composite index

The cumulative doses of adriamycin at baseline and in early, interim and later stages were 0, 100, 200, and 300 mg/m^2^, respectively. Spearman correlation analysis showed that the cumulative dose of adriamycin was positively correlated with GCS, GLS and MCI, and negatively correlated with GRS and LVtw. With the increase of cumulative dose of adriamycin, all absolute values of GRS, GCS, GLS, LVtw and MCI were decreased (Table 3).

**TABLE 3:** Analysis on the correlations of the cumulative dose of adriamycin with GRS, GCS, GLS, LVtw, and MCI.

Item	*r*	*P*
GRS	–0.414	0.000
GCS	0.428	0.000
GLS	0.627	0.000
LVtw	–0.741	0.000
MCI	0.760	0.000

### Evaluation of effectiveness of two-dimension speckle tracking echocardiography parameters and myocardial composite index in the prediction of subclinical dysfunction

**FIGURE 2:**
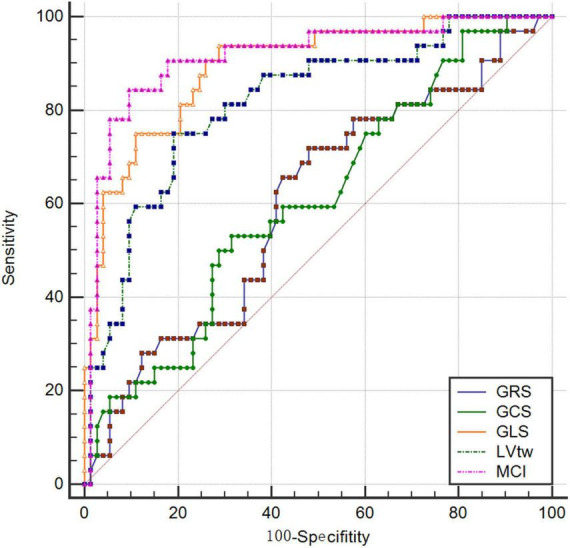
ROC curve for 2D-STE parameters and MCI.

The results of this study showed that the AUCs of GRS, GCS, GLS, LVtw and MCI for subclinical LV dysfunction prediction were 0.607, 0.601, 0.893, 0.814, and 0.915, respectively, and their corresponding sensitivities were 53.13%, 71.87%, 93.75%, 75.00% and 84.37%, respectively, and their corresponding specificities were 68.49%, 52.05%, 71.23%, 80.82% and 90.41%, respectively (Table 4 and Figure 2).

**TABLE 4:** Results of ROC analysis of 2D-STE parameters and MCI.

Indexes	AUC	Standard error	*P*	95%*CI*	Cutoff value	Sensitivity (%)	Specificity (%)
GRS (%)	0.607	0.0595	0.096	0.507–0.701	44.91	53.13	68.49
GCS (%)	0.601	0.0608	0.073	0.501–0.696	–17.73	71.87	52.05
GLS (%)	0.893	0.0339	<0.001	0.818–0.945	–17.06	93.75	71.23
LVtw (°)	0.814	0.0463	<0.001	0.727–0.883	12.97	75.00	80.82
MCI (:%×°)	0.915	0.0328	<0.001	0.845–0.961	–210.89	84.37	90.41

## Discussion

### Anthracycline-induced cardiotoxicity and its mechanism

Anthracycline-induced cardiotoxicity mainly causes changes in cardiac electrophysiology and cardiac hemodynamics, which are manifested as abnormal ECG, decreased LVEF and abnormal myocardial activity, and may eventually lead to heart failure.

The pathological manifestations of the damaged myocardium caused by anthracycline toxicity mainly include myocardial edema, disappearance of myocardial cells, interstitial fibrosis and sarcoplasmic reticulum expansion under light microscope, and myocardial fibrinolysis, extensive disappearance of fiber bundles, deformed and broken Z-line, mitochondrial lysis and vacuole formation in myocardial cells under the electron microscope (4).

The cardiotoxicity of anthracycline anticancer drugs can be divided into the following three types according to its onset time and progression speed: (1) acute or subacute cardiotoxicity which usually occurs within 2 weeks after the start of treatment; (2) chronic cardiotoxicity which usually occurs within 1 year after the completion of chemotherapy and is the most common clinical cardiotoxicity; (3) delayed cardiotoxicity which is cardiac injury occurring at 1 year after completion of chemotherapy (5).

The exact mechanism of anthracycline-induced cardiotoxicity remains unclear. At present, the main categories of views are as follows: (1) The most classic oxidative stress theory: Anthracycline drugs can generate oxidative active substances such as superoxide anions through a series of processes in the mitochondria of myocardial cells, which can lead to apoptosis and necrosis of myocardial cells. Meanwhile, anthracycline drugs can lead to a failure of myocardial cells to metabolize harmful substances such as oxygen free radicals in time and further aggravate the myocardial injury (6). Anthracycline drugs can increase the iron content in myocardial cells, leading to the production of hydroxyl radicals, which in turn damage mitochondrial DNA (7). (2) Calcium overload theory: Anthracycline drugs can rapidly increase the intracellular free Ca^2+^ content, which can inhibit the excitation-contraction coupling of myocardial cells and induce arrhythmias (8).

### Evaluation of cancer therapeutics-related cardiac dysfunction and subclinical LV dysfunction

Endocardial myocardial biopsy is recognized as the gold standard for evaluating the anthracycline-induced cardiotoxicity, but this method is invasive and rarely used in clinical practice. Therefore, LVEF is generally used to evaluate cardiac dysfunction related to cancer treatment in clinical practice. American Society of Echocardiography (ASE) and European Association of Cardiovascular Imaging (EACVI) defines the cancer therapeutics-related cardiac dysfunction (CTRCD) as: the decrease in LVEF caused by cancer treatment is > 10% of baseline LVEF and reaches less than 53% absolute value (9). In this study, finally it was found that 5 patients finally met the diagnostic criteria for cancer therapeutics-related cardiac dysfunction (CTRCD). However, LVEF has some limitations in monitoring cardiotoxicity. LVEF is a load-dependent index, which is easily affected by preload, afterload and rhythm of the heart. At the same time, the chemotherapy-induced cardiotoxicity is regional, and some myocardial segments may compensate for the loss of function of other myocardial segments, thus maintaining a normal LVEF level at least in the early stage (10). Moreover, this is credible only if the difference in LVEF measured by 2D echocardiography among paitents in different stages reaches 10%. However, this is the same magnitude of change used to adjudicate CTRCD. Therefore, scholars doubt the sensitivity of LVEF measured by 2D echocardiography in diagnosing CTRCD (9).

In addition, many studies have shown that the myocardium has been injured before the abnormal LVEF occurs (2), and the myocardial injury has been irreversible at that time (1), so that the timing for cardioprotective intervention is delayed. Therefore, LVEF is not sensitive for early detection of subclinical heart disease, and it cannot sensitively reflect the small changes in left ventricular function.

Based on this, clinicians have been trying to diagnose this latent subclinical myocardial injury before LVEF is significantly reduced in clinical practice. At present, the imaging diagnosis methods of subclinical LV dysfunction mainly contain various new ultrasound technologies such as velocity vector imaging, strain rate imaging, tissue Doppler imaging and STE, of which, the STE technique (mainly including 2D-STE and 3D-STE techniques) is not affected by the angle of sound beam and thus can be used to perform quantitative analyses, which can also be visually displayed with the “bull’s eye diagram” at the same time, it has the characteristics of sustainable dynamic detection and low cost, shows increasing obvious advantages and has become a research hotspot in recent years. The main observation indexes of STE technique include parameters such as longitudinal strain, radial strain, circumferential strain, area strain, twist and synchronization of left ventricular myocardium. The STE technique is not only used to analyze the basal segment, the middle segment and the apical segment of the standard section separately, but also used to stratifiedly analyze the epimyocardium, mid-myocardium and endomyocardium of the left ventricular wall. It is recommended by the consensus committee consisting of ASE and EACV that a relative reduction of more than 15% in GLS from baseline in patients receiving anticancer therapy can be used as a diagnostic criterion for subclinical LV dysfunction (9). In this study, finally it was found that 22 patients met the diagnostic criteria for subclinical LV dysfunction.

### Assessment of anthracycline -induced cardiotoxicity by global longitudinal strain, left ventricular twist and myocardial composite index

Many studies have shown that GLS is considered to be the most sensitive marker of subclinical LV dysfunction in the application of 2D-STE (11–14), and there are mainly the following two views on its pathological basis: (1) According to Torrent-Guasp’s myocardial band theory, the cardiac pulsation is composed of a complex towel-wringing-like movement of the myocardium; and (2) the endomyocardium is mainly composed of longitudinal myocardial fibers responsible for longitudinal movement, and the endocardium is more vulnerable to damage due to its direct contact with chemotherapy drugs. In this study, there was a difference in GLS between patients in the interim chemotherapy stage and at baseline, and GLS was decreased gradually with the increase of the cumulative dose of adriamycin, which was similar to previous research results at home and abroad.

This study also showed that LVtw also presented a good diagnostic value, and there was a significant difference in LVtw between patients in the interim chemotherapy stage and at baseline, which was decreased continuously with the increase of the cumulative dose of adriamycin. An in vitro study has confirmed that there are two main mechanisms for the decrease in twist mechanics in patients treated with anthracycline drugs. First, Anthracycline drugs can cause myofilament degradation by activating calpain, resulting in myofilament disorder. Carnosine is the largest known protein and a component of myofilament system, and its effect on the activation and recovery of cardiomyocytes has been confirmed. Second, it has been reported that twist mechanics is closely related to the transmural gradient of carnosine subtypes in animal models (15).

Systole and diastole of left ventricle are a complex movement process in 3D space, which can be divided into the longitudinal, radial and circumferential movements, and its own torsion movement at the same time. MCI can comprehensively analyze the movements in both directions of left ventricular twist and longitudinal strain at the same time. Previous studies have shown that MCI has a better diagnostic efficacy compared with the index in single direction for early cardiotoxic injury (11–13, 16). In this study, there were no differences in GLS and LVtw between patients in the early chemotherapy stage and at baseline, but MCI combining two parameters such as GLS and LVtw showed a difference between patients in the early chemotherapy stage and at baseline. Meanwhile, MCI showed a trend of continuous worsening with the increase of chemotherapy cycles, indicating that MCI was related to the cumulative dose of adriamycin. Moreover, the area under the curve (AUC) of MCI was largest after ROC analysis, and its diagnostic efficacy was higher than those of other parameters in a single direction.

In a previous study on the latest 3D-STE technique, the global area strain (GAS) has been used as a new index, which simultaneously integrates the longitudinal and circumferential strains of the myocardium (17). Compared with the strain in a single direction, the GAS is superior to the conventional strain parameters in detecting early left ventricular systolic dysfunction (18, 19).

At present, there are few research reports on MCI, and more research data are needed to evaluate the subclinical LV dysfunction. In addition, MCI needs to be calculated manually. Subsequently, if the echocardiography manufacturers can realize the objective of directly calculating this index in the analysis software used for the STE technique, the convenience of operation will can be improved.

### Clinical significance

This study shows that STE technique is of great significance in identifying early myocardial injury, and can help clinicians predict the cardiotoxicity of anthracycline in the treatment of breast cancer patients earlier. Because the cardiac pulsation is a complex 3D movement, MCI can comprehensively analyze the movements of the heart in the multi-dimensional space, and can detect the subclinical cardiac injury earlier. This index can help clinicians timely participate in the cardioprotective treatment, control the accumulated dose of chemotherapy drug and reduce the cardiotoxicity of drugs to avoid the irreversible myocardial injury.

## Limitations

The limitations of this study were as follows: (1) The sample size of this study was small, and we will conduct a relative study with a larger sample size in the future; (2) the patients in this study were followed up for a short period of time, and the long-term effects of chemotherapy drugs on various cardiac parameters could not be observed; (3) biomarkers such as troponin were not included into this study, and these indexes can be included into subsequent study; and (4) the selected patients were not analyzed using 3D-STE technique, and GAS was not included in the comparative analysis, which will be analyzed in the future studies.

## Conclusion

STE technique can detect anthracycline-induced myocardial injury in time; compared with 2D-STE parameters in a single direction (GLS, GCS, GRS, and LVtw), MCI can detect subclinical LV dysfunction earlier and has a better diagnostic efficiency; the patients receiving anthracycline chemotherapy may have subclinical LV dysfunction even if their LVEF is normal, and the subclinical LV dysfunction is manifested as deteriorated GLS, LVtw, and MCI, which are correlated with the cumulative dose of anthracycline drugs.

## Data availability statement

The original contributions presented in the study are included in the article/supplementary material, further inquiries can be directed to the corresponding author.

## Ethics statement

The study was approved by the ethics committee of Nantong Third People’s Hospital. The patients/participants provided their written informed consent to participate in this study.

## Author contributions

JZ and JW designed the study. HJ, JZ, SX, and XG contributed to data collection, analyses and interpretation. JZ contributed to the statistical analysis. HJ, JZ, and JW contributed to the manuscript writing, reviewing and editing. All authors read and approved the final manuscript.
